# Murine model of cross-IgE sensitization and cross-anaphylactic reactions among multiple group food allergens

**DOI:** 10.3389/fimmu.2024.1497368

**Published:** 2025-01-07

**Authors:** Ibrahim Musa, Fariba Ardalani, Nan Yang, Soheila J. Maleki, Xiu-Min Li

**Affiliations:** ^1^ Department of Pathology, Microbiology & Immunology, New York Medical College, Valhalla, NY, United States; ^2^ General Nutraceutical Technology LLC, Elmsford, NY, United States; ^3^ United States (U.S.) Department of Agriculture, Agriculture Research Service, Southern Regional Research Center, New Orleans, LA, United States; ^4^ Department of Otolaryngology, School of Medicine, New York Medical College, Valhalla, NY, United States; ^5^ Department of Dermatology, School of Medicine, New York Medical College, Valhalla, NY, United States

**Keywords:** multiple food allergy murine model, cross IgE sensitization, cross anaphylactic reactivity, allergen specific IgE, allergen specific IgG subclass antibodies

## Abstract

**Rationale:**

Approximately 32 million people in the United States suffer from food allergies. Some food groups, such as legumes – peanuts, tree nuts, fish, and shellfish, have a high risk of cross-reactivity. However, the murine model of multiple food group cross-reactivity is limited.

**Objective:**

We sought to develop a murine model that can be used to investigate novel therapeutics for the treatment of multiple food allergies.

**Methods:**

C3H/HeJ mice were sensitized intraperitoneally (i.p.) once a week for three weeks with a mixture of 500µg of protein from peanut, cashew, walnut, shrimp, cod, and 2 mg Alum. The control group consisted of naïve mice. IgE levels against the sensitized allergens and their cross-reactive allergens were measured by ELISA at baseline and 3 weeks after sensitization. In weeks 4 and 5, the mice were given intragastric challenges with 200mg/mouse of each food: peanut, chickpea, lentil, cashew, almond, pistachio, hazelnut, brazil nut, walnut, pecan, shrimp, lobster, cod, salmon, and mackerel. After each challenge, anaphylactic symptoms, rectal temperatures, and plasma histamine were measured.

**Results:**

There was a significant elevation of IgE against sensitized antigens (peanut cashew, walnut, shrimp, and cod) as well as cross-reactive allergens used for oral food challenge from legumes including peanut, chickpea, and lentil, as well as tree nuts such as cashew, almond, pistachio, hazelnut, brazil nut, walnut, and pecan. Furthermore, there was a significant increase in crustaceans such as shrimp, lobster and fish like cod, salmon, and mackerel (*p*<0.01). Consistently, significantly increased anaphylactic symptom scores (*p*<0.05), decreased rectal temperature (*p*<0.001), and increased plasma histamine (*p*<0.05) compared to the naïve mice occurred following each challenge with sensitized foods and unsensitized, but cross-reactive foods.

**Conclusion:**

We generated a comprehensive murine model of IgE-mediated multiple food groups of cross-reactive anaphylaxes. This will provide an essential tool for developing novel therapies for cross-reactivity multiple food allergies.

## 1 Introduction

In the United States, approximately 32 million people are affected by food allergies, with 8% of children and 11% of adults being impacted. Cross-reactivity refers to the recognition of secondary allergens by IgE antibodies or other immune responses that have previously been sensitized to a primary allergen. This occurs when the primary allergen and the secondary allergen share regions or sequences of amino acids, known as epitopes (1). Cross-reactivity in food allergens is thought to be triggered by a 70% similarity in the amino acid sequence (2), however, the structure and overall physiochemical composition of the allergens and the epitopes are the primary factors behind cross-reactivity and allergies (3, 4). Food allergens often resist the breakdown processes of the digestive system, leading to the allergen structure being identified by the gastrointestinal tract’s immune system (5, 6). Food allergens that remain intact in the digestive system are recognized and taken up by specialized cells called M cells (7). Furthermore, mucosal dendritic cells can capture these proteins and present them to MHC class II molecules, which ultimately activate naïve T cells (8).

The question of whether people allergic to peanuts should avoid other legumes often arises in clinical practice. *In vitro*, serologic cross-reactivity has demonstrated a connection between peanut and allergens in most other legume species, including lupine, soybean, and green pea (9). Peanut and tree nut allergies frequently occur in the same patients, with a rate of 30% to 50% in some study groups. However, the specific tree nut reactivity patterns vary. Most studies have reported on tree nut sensitization rather than challenge-proven allergy (10). For example, the Australian Health Nuts Study, which was a prospective study on the development of peanut allergy, showed that 61% of 4-year-olds with a peanut allergy were sensitive to tree nuts such as cashews, almonds, or hazelnuts (11). In another study involving 234 children with a peanut allergy, 86% were found to be sensitive to tree nuts. Out of these, 34% were confirmed to have a clinical allergy to at least one of the tree nuts, despite most of them never having consumed any tree nuts (12).

Seafood can be divided into two main categories: shellfish and fish. Examples of shellfish include crustaceans such as shrimp and lobster and mollusks such as snails and squids. Fish can be categorized as either bony, such as salmon and cod, or cartilaginous, such as sharks and rays. The risk of cross-reactivity between these two main groups of seafood is low because the major allergen in fish (parvalbumin) is different from the major allergen in shellfish (tropomyosin) (13, 14). Several studies have shown that there is a low rate of cross-reactivity between the shellfish and fish group. US prevalence studies (15) have reported that 10% of those with either fish or shellfish allergy have reported allergies to both. A retrospective study noted that at least 21% of 167 children with seafood allergy reported allergies to both fish and shellfish (16). Nevertheless, cross-reactivity within each group (fish and shellfish) remains a concern.

Different mouse models are used in food allergy studies, each serving specific research purposes. BALB/c mice are commonly utilized in allergy research because of their Th2-skewed immune response, which makes them more susceptible to developing IgE-mediated reactions. These mice typically show strong responses to allergens, characterized by elevated levels of IgE and the production of cytokines like IL-4 and IL-13. BALB/c mice are well-suited for investigating the mechanisms of sensitization, IgE production, and the role of Th2 responses in food allergies. However, they may not display severe anaphylactic symptoms, which can limit their effectiveness in researching extreme allergy cases. FcϵRI-deficient mice lack the high-affinity IgE receptor (FcϵRI) on mast cells and basophils, which play a crucial role in IgE-mediated allergic reactions. These mice are used to study the roles of IgE and FcϵRI signaling in food allergies, as well as to test interventions aimed at blocking these pathways. However, since they do not naturally exhibit IgE-mediated reactions, their utility in investigating spontaneous allergic symptoms is restricted. OVA-sensitized models frequently use ovalbumin (OVA) as a model allergen in food allergy studies. This model is useful for examining basic immunological responses and mechanisms of tolerance. However, because OVA does not fully mimic human food allergens, its relevance for direct clinical application is limited. The C3H/HeJ mouse model was selected for this study due to its susceptibility to anaphylactic reactions when exposed to food allergens. These mice display significant IgE responses and symptoms of anaphylaxis, such as a drop in core body temperature. This model effectively simulates cross-reactivity and allergies to multiple food groups, aligning well with the main objectives of the study. C3H/HeJ mice are particularly valuable for assessing the severity of allergic responses across various food allergens and for developing potential treatments for cross-reactive food allergies, given their robust reactions to allergen exposure Yang et al. (20).

Therefore, we conducted this study to create a model to determine if the cross-reactivity *in vitro* can be seen *in vivo*. Cross-reactivity has been demonstrated in some foods to which individuals are allergic, but there has been no demonstration of clinical allergy. The only clinical demonstration of the allergy is by oral food challenge, which has some potential risks. Therefore, we created a murine model that can be used to study cross-reactivity *in vivo*. Avoidance is not the only form of management care for cross-reactive food allergens. This model will serve as a tool that can be used to develop new therapeutics to prevent and manage multiple food allergies.

## 2 Materials and methods

### 2.1 Protein extraction

To prepare food extracts, legumes such as peanuts (PN), chickpeas (CHKP), and lentils (LNTL) were ground and defatted using acetone and then homogenized in PBS, as previously described (17). Tree nuts like cashew (CSH), almond (ALM), pistachio (PIST), hazelnut (HZN), brazil nut (BZN), walnut (WLN), pecan (PCN) and macadamia (MCDM), as well as crustaceans such as shrimp (SHR), lobsters (LOB), fresh cod filets (COD), salmon (SAL), and mackerel (MACK), were bought from local markets (Shop Rite, Westchester, NY), defatted and homogenized in PBS. The process of protein extraction commenced with the thorough homogenization of peanut, chickpea, lentil, cashew, almond, pistachio, hazelnut, brazil nut, walnut, pecan, shrimp, lobster, cod, salmon, and mackerel using a blender. This resulted in the formation of a blended slurry. Subsequently, the homogenized material underwent defatting with acetone using a magnetic stirrer, and the resulting solution was filtered using a funnel. This sequence of steps was iterated until the acetone achieved a state of transparency. Following this, the defatted food extract was collected and subjected to an overnight drying process under a laminar flow hood to ensure the complete removal of acetone and the attainment of thorough material drying. The protein extraction was done by dissolving the defatted food powder in PBS. This mixture was then subjected to stirring at a temperature of 4°C for a duration of 2-6 hours. Finally, the extraction process culminated in the acquisition of the crude protein extract through a centrifugation process operating at 3,000 rpm for a timeframe of 30 minutes at 4°C, with the subsequent collection of the supernatant. The protein concentrations of the extracts were measured using the BioRad protein assay reagent kit (Bio-Rad Laboratories) (18) See **Table 1**.

**Table 1:** Concentration in mg/mL of food extract used for intraperitoneal sensitization and ELISA.

Priming Allergen			Cross reactive Allergen		
Family	Protein	Concentration for IP and ELISA (mg/mL)	Family	Protein	Concentration for ELISA (mg/mL)
Legumes	Peanut	14.8	Legumes	Chickpea	17.95
Treenuts	Cashew	16.65		Lentil	22.38
	Walnut	18.51	Treenuts	Almond	19.79
Crustacean	Shrimp	25.4		Macadamia	21.05
Fish	Cod	19.25		Pistachio	25.95
				Brazilnut	23.2
				Hazelnut	19.85
				Pecan	28.45
			Crustacean	Shrimp	26.4
			Fish	Salmon	25.55

The protein concentrations of the extracts were measured using the BioRad protein assay reagent kit.

### 2.2 Mice

**Figure 1:**
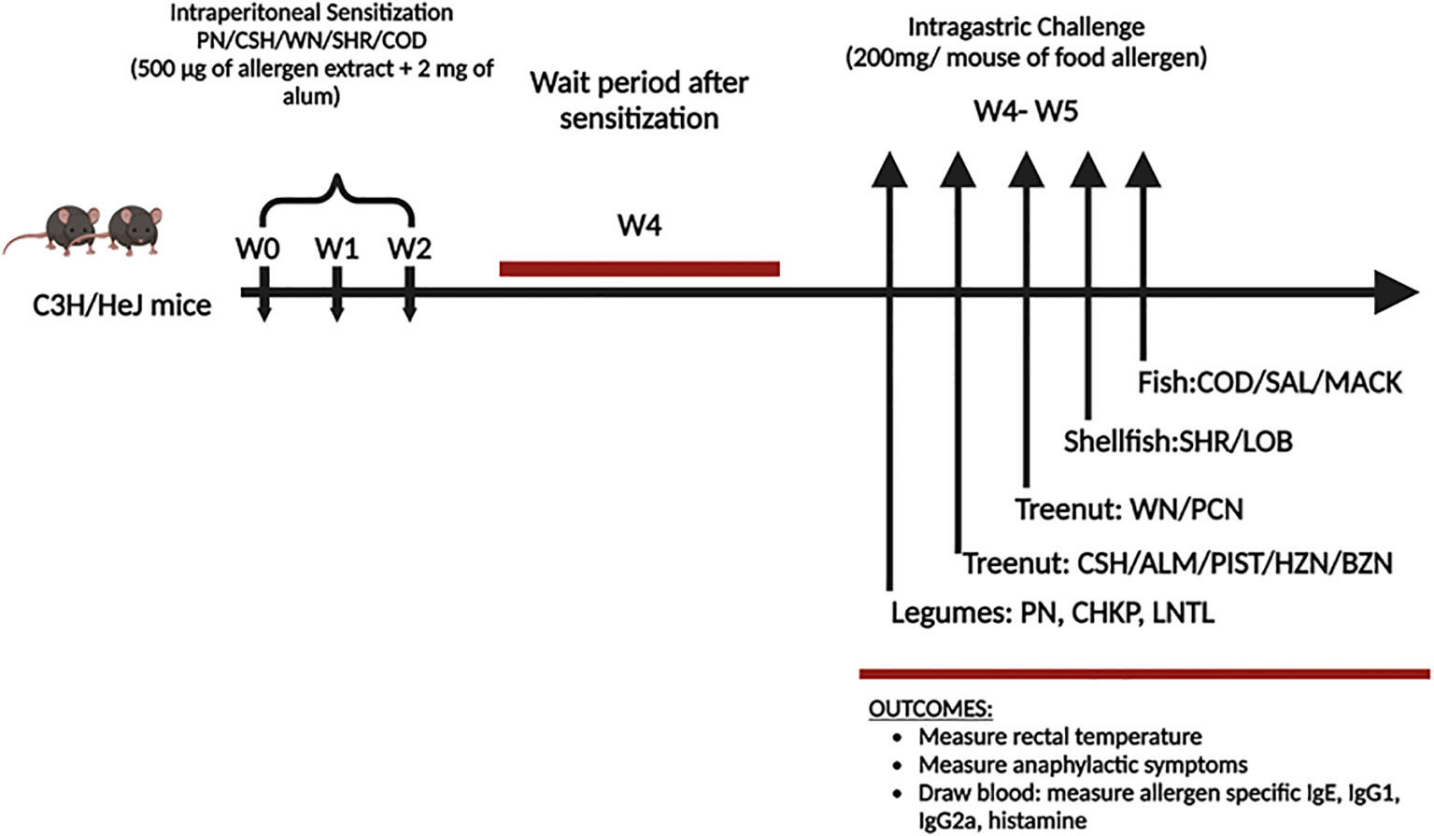
Experimental protocol. C3H/HeJ mice (n=15/group) weresensitized with peanut (PN), cashew (CSH), walnut (WN), shrimp (SHR) and Cod (500µg each food protein extract) + 2 mg alum intraperitoneally (I.P.) weekly for 3 weeks (w0-w2). Blood was drawn before the challenge (w4). At weeks 4-5, mice received intragastrical (i.g.) challenges (200 mg/mouse of extract): Legumes such as Peanut (PN) in day 1, Chickpea (CHKP), and Lentil (LNTL) in days 2-3. Tree nuts include Cashew (CSH), Brazil nut (BZN), Almond (ALM), Pistachio (PIST), Pecan (PCN), Macadamia (MCDM), Hazelnut (HZN), and Walnut (WN) in days 3-8. Fish such as Cod (COD), Salmon (SAL), and Mackerel (MACK) in days 9-11. Crustaceans such as Shrimp (SHR) and Lobsters (LOB) in days 12-14.

In previous studies by Kulis et al. (19), C3H/HeJ mice were sensitized through intraperitoneal injection of cashew alone (monosensitized mice) or cashew plus walnut, using alum as an adjuvant. Both groups were then exposed to challenges involving cashews, walnuts, and peanuts, and subsequent monitoring was conducted to observe anaphylactic reactions. Anaphylactic antibodies were quantified using ELISA (19). Similarly we purchased 30 female C3H/HeJ mice of six weeks of age from the Jackson Laboratory in Bar Harbor, ME, to study food allergy. These mice are susceptible to oral anaphylaxis, making them ideal for such studies. The mice were kept in a specific pathogen-free environment and fed allergen-free chow, following standard animal care and use guidelines. The study included two groups, one with 15 mice sensitized to allergens and the other with 15 naive controls. See **Figure 1**. All animal experiments conducted in this study was approved and carried out in strict compliance with the regulations and protocols set forth by the Institutional Animal Care and Use Committee (IACUC) of New York Medical College IACUC approval # 15156 and #20857.

### 2.3 Cross-reactive allergen sensitization, boosting, and challenge

To create a cross-reactive allergy mice model, we sensitized the mice intraperitoneally once per week for three weeks with 500mg of crude PN, CSH, WAL, SHR, COD extract, and 2mg of alum in 200uL of phosphate-buffered saline (PBS) purchased from ThermoFisher Scientific (Waltham, MA). The naive group received PBS alone as sham sensitized (sham). We collected serum for allergen-specific immunoglobulin E (IgE) measurement three weeks following the sensitization. In the next step, we challenged the mice in the sensitized and the naïve group intragastrically with 200mg of freshly ground PN, CHKP, LNTL, CSH, ALM, PIST, HZN, BZN, WAL, PCN, SHR, LOB, COD, SAL, MACK in 500uL PBS after the last sensitization.

### 2.4 Measurement of allergen-specific IgE, IgG1, IgG2a levels

Blood was collected from the submandibular vein before the challenge. The collected sera were then stored at -80°C until analysis. Following a previously described protocol (20), allergen-specific IgE, IgG1, and IgG2a levels were determined using a monoclonal antibody. ELISA plates were coated with protein extracts PN, CHKP, LNTL, CSH, ALM, PIST, HZN, BZN, WAL, PCN, SHR, LOB, COD, SAL, MACK in the sample wells, as well as anti-mouse IgE for IgE reference wells 2,4-Dinitrophenyl-Human Serum Albumin (DNP-HSA) (for IgG1 reference wells and IgG2a reference wells). After coating the plates, they were incubated overnight at 4°C. The next day, the plates were washed and blocked with 2% BSA-PBS. They were then incubated overnight at 4°C with serum samples that were diluted in the incubating buffer, mouse IgE (BD Biosciences), and anti-DNP-IgG1, anti-DNP-IgG2a (Accurate Antibodies, Westbury, NY). On the following day, the plates were washed again and incubated with biotinylated anti-IgE IgG2a or IgG1 detection antibodies (BD Biosciences),avidin-peroxidase, and ABTS substrate (KPL, St. Paul, Minn). Finally, the optical density was read using a microplate reader at a wavelength of 405nm.

### 2.5 Assessment of hypersensitivity reactions and measurement of rectal temperature

The type 1 hypersensitivity reactions were assessed at intervals of 10 minutes for one hour after the challenge. To evaluate anaphylactic symptom scores, we referenced studies by Srivastava et al. (21) which investigated peanut oral immunotherapy using CpG/peanut nanoparticles in a murine model of peanut allergy. The anaphylactic symptom scores were assigned based on the severity of symptoms as follows: 0: No signs, 1: Scratching and rubbing around the nose and head, 2: Puffiness and redness around the eyes and mouth, diarrhea, piloerection, reduced activity, and/or increased respiratory rate, 3: Wheezing, labored respiration, and cyanosis around the mouth and tail, 4: Symptoms consistent with score 3, accompanied by no activity after prodding, tremors, or convulsions, 5: Death. Rectal temperatures were measured every 10 minutes for 120 minutes after the peanut challenge using a thermal probe (Harvard Apparatus, USA).

### 2.6 Plasma histamine, a measure of mast cell degranulation

Thirty minutes after the challenge, plasma was collected, and the levels of histamine were determined using an enzyme immunoassay kit (ImmunoTECH, Marseille, France), as previously described (22). (Immunotech, France and Moredun Scientific, UK for histamine).

### 2.7 Statistics

All statistical analyses were performed using GraphPad Prism (version 9, GraphPad Software, Inc, San Diego, CA). We conducted one-way ANOVA (analysis of variance) and then applied the Bonferroni correction for all pairwise comparisons. In cases where the data was skewed, we used one-way ANOVA on rank to assess the differences between groups and then applied Donne’s method for all pairwise comparisons. We used a two-sided test to calculate p-values for all tests, and a p-value of ≤ 0.05 was considered statistically significant.

## 3 Results

### 3.1 Induction of anaphylaxis following the challenge of the initially sensitized foods

In designing our study, we referenced previously published protocols. One study conducted by Li et al. described a murine model of peanut anaphylaxis, demonstrating that T- and B-cell responses to a major peanut allergen closely mimic human response (23). Another study by Pons et al. explored soy immunotherapy for peanut-allergic mice, focusing on modulating the peanut-allergic response (24). In their research, the sensitized group of C3HeJ mice received allergen along with alum as an adjuvant, while the naïve controls were given PBS. We adopted this method, sensitizing C3HeJ mice with allergen and alum, while the naïve controls received only PBS. We used 30 female C3HeJ mice that were 6 weeks old to create a model of multi-food allergies in mice. 15 mice were sensitized for 3 weeks by injecting them with 500µg of peanut, cashew, walnut, shrimp, cod protein, and 2 mg of alum. In the fourth week, we drew blood from the submandibular facial veins of the mice to measure serum allergen-specific Immunoglobulin E (IgE). In the fifth week, the sensitized mice underwent an intra-gastric sequential oral food challenge using 200mg of PN, CHKP, LNTL, CSH, ALM, PIST, HZN, BZN, WAL, PCN, MCDM SHR, LOB, COD, SAL, MACK. We measured anaphylactic symptom scores every 10 minutes for 60. Rectal temperature was measured every 10 minutes following the oral food challenge, and plasma was collected to measure histamine levels using ELISA (**Figure 1**).

### 3.2 Induction of peanut-, cashew-, walnut-, shrimp- and cod-specific IgE following systemic sensitization

**Figure 2:**
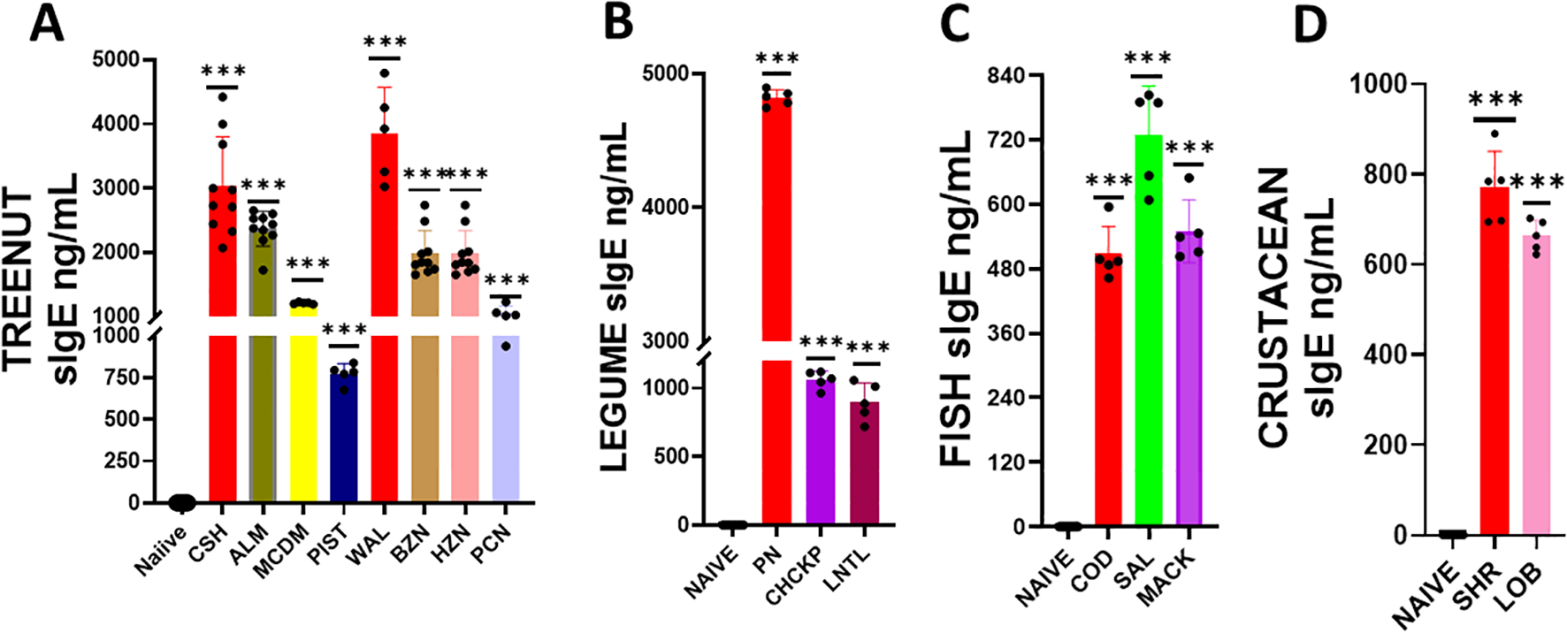
Serum Allergen-specific IgE: Sera were harvested after blood collection by submandibular bleeding at the indicated time points. Serum allergen-specific IgE levels for **(A)** Tree nuts, **(B)** Legumes, **(C)** Fish, and **(D)** Crustaceans were determined by ELISA. Inoculated foods have red solid bars. ***p< 0.001 vs sham. n=10-15 mice/group.

We determined if serum Immunoglobulin E (IgE) produced against sensitized protein would cross-react with other allergens (**Figure 2**). We coated ELISA plates with proteins derived from the following allergic foods: peanut, chickpea, lentil, cashew, almond, pistachio, hazelnut, brazil nut, walnut, pecan, shrimp, lobster, cod, salmon, and mackerel. We added serum from mice sensitized with the previously specified food allergens (peanuts, cashews, walnuts, shrimps, and cod), to the coated plates and measured the allergen-specific IgE. In the tree nut group, the IgE levels against the sensitized allergens were as follows: cashew IgE ranged between 2065-4419 ng/mL, and Walnut ranged between 3019-4790 ng/mL. The levels of cross-reactive IgE to other tree nuts allergens were as follows: brazil nut ranged 1647-2731 ng/mL, almond ranged 1719 – 2648 ng/mL, pistachio ranged between 675 – 838 ng/mL, pecan ranged between 938 – 1225 ng/mL, macadamia ranged between 1186-1223 ng/mL, and hazelnut ranged between 1647 – 2731 ng/mL (**Figure 2A** ***p< 0.001 vs sham). Similarly, in the legume food group, sensitized against peanuts had IgE levels in the range between 4746 – 4894 ng/mL, while the cross-reactive allergens chickpea and lentils had IgE levels ranged between 964-1119 ng/mL and 718 – 1057 ng/mL, respectively (**Figure 2B** ***p< 0.001 vs sham). In the fish food group, sensitized against cod allergen had IgE levels in the range of 462– 595 ng/mL, while the cross-reactive allergens salmon and mackerel had IgE levels ranged between 607- 789 ng/mL and 502 – 649 ng/mL, respectively (**Figure 2C** ***p< 0.001 vs sham). For the shellfish food group, sensitized allergen shrimp had IgE levels in the range of 694– 889 ng/mL, while the cross-reactive allergen lobster had IgE levels ranging between 622-702 ng/mL (**Figure 2D** ***p< 0.001 vs sham).

### 3.3 Induction of peanut-, cashew-, walnut-, shrimp- and cod-specific IgG1 and IgG2a following systemic sensitization

**Figure 3:**
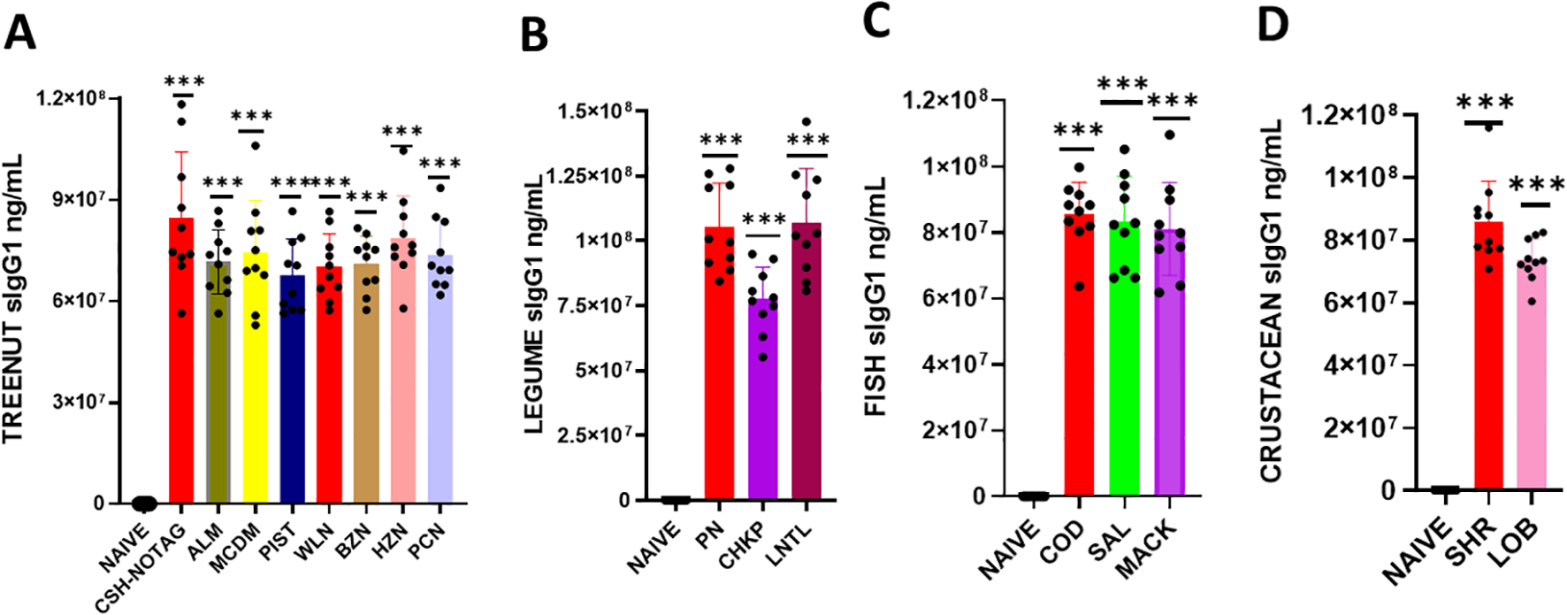
Serum Allergen-specific IgG1: Sera were harvested after blood collection by submandibular bleeding at the indicated time points. Serum allergen-specific IgG1 levels **(A)** Tree nuts, **(B)** Legumes, **(C)** Fish, **(D)** and Crustaceans, were determined by ELISA. Inoculated foods have red solid bars. ***p< 0.001 vs sham. n=10-15 mice/group.

**Figure 4:**
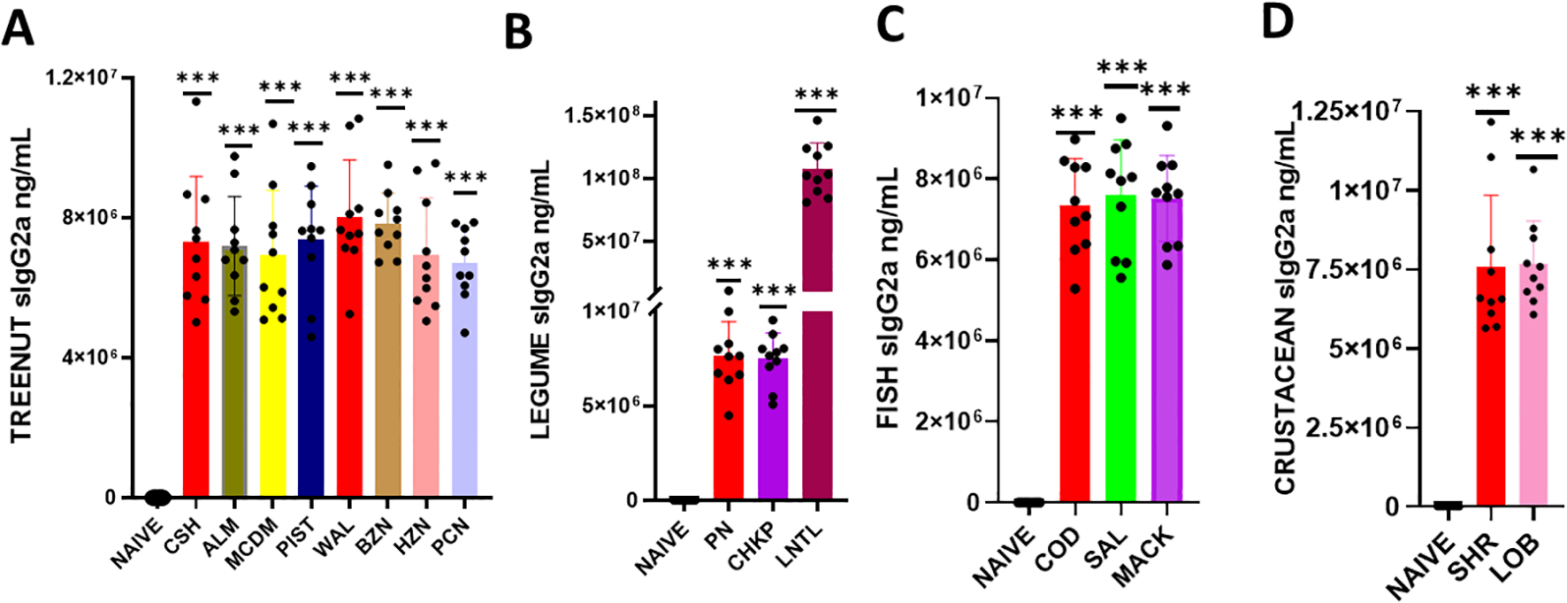
Serum Allergen-specific IgG2a: Sera were harvested after blood collection by submandibular bleeding at the indicated time points. Serum allergen-specific IgG2A levels **(A)** Tree nuts, **(B)**, Legumes **(C)**, Fish **(D)**, and Crustaceans were determined by ELISA. Inoculated foods have red solid bars. ***p< 0.001 vs sham. n=10-15 mice/group.

We determined whether serum Immunoglobulin G1 (IgG1) and G2a (IgG2a) produced in response to a sensitized protein would exhibit cross-reactivity with other allergens (**Figures 3**, **4**). To do this, we coated ELISA plates with proteins similar to the ones mentioned above, and using ELISA, we assessed the reactivity of IgG1 and IgG2a against various food allergens. Our findings showed that the levels of IgG1 and IgG2a in the tree nut group were significantly higher than in the naïve group (**Figures 3A, B** * *p*< 0.05; ** *p*< 0.01; ***p< 0.001 vs sham). Similarly, in the legume, fish, and crustacean food groups, the levels of IgG1 and IgG2a were significantly elevated compared to the naïve group (**Figures 3B–D**, **4B, D** * *p*< 0.05; ** *p*< 0.01; ***p< 0.001 vs sham).

### 3.4 Cross-reactive food allergens induced decreased rectal temperature

**Figure 5:**
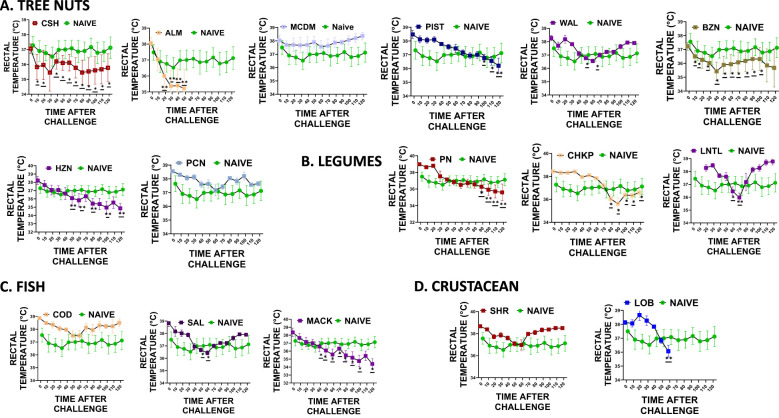
Rectal Temperature. At weeks 4-5, mice received intragastrical (i.g.) challenges (200 mg/mouse of extract) as follows: **(A)** Tree nuts such as Cashew, Almond, Macadamia, Pistachio, Walnut, Brazil nut, Hazelnut, and Pecan. **(B)**. Legumes such as Peanut, Chickpea, and Lentil. **(C)** Fish such as Cod, Salmon, and Mackerel **(D)**. Crustaceans such as Shrimp and Lobsters. Rectal temperatures were assessed every 10 mins till 120 mins following each food challenge. *p< 0.05; **p< 0.01; ***p< 0.001 vs sham. n=10-15 mice/group.

During each intragastric food challenge, we measured the rectal temperature of mice after administering 200mg of freshly prepared chickpea, lentil, almond, pistachio, hazelnut, brazil nut, pecan, lobster, salmon, and mackerel to both the naïve and sensitized groups (**Figure 5**). We measured the rectal temperature of mice every 10 minutes for 120 minutes after administering the food allergen intragastrically. Our findings revealed that the sensitized mice that received oral peanut food allergens exhibited significantly lower rectal temperatures than the naïve mice that did not undergo sensitization (**Figure 5B** * p< 0.05; ** p< 0.01; ***p< 0.001 vs. sham). Additionally, mice that received cross-reactive food allergens from the legume food group, such as chickpea and lentil, also displayed significantly lower rectal temperatures than the naïve group (**Figure 5B** * *p*< 0.05; ** *p*< 0.01; ***p< 0.001 vs sham). Similar trends were observed in the tree nut-sensitized group, where sensitized mice exposed to cashews and walnuts showed significantly lower rectal temperatures than naïve mice. Furthermore, mice exposed to cross-reactive tree nuts allergens like brazil nut, almond, pistachio, pecan, and macadamia also demonstrated significantly lower rectal temperatures (**Figure 5A** * p< 0.05; ** p< 0.01; ***p< 0.001 vs. sham). The fish and crustacean group also displayed similar patterns, with mice exposed to cod and shrimp allergens showing significantly lower rectal temperatures than the mice exposed to salmon, mackerel, and lobster (**Figures 5C, D** * *p*< 0.05; ** *p*< 0.01; ***p< 0.001 vs sham).

### 3.5 Cross-reactive allergen-induced anaphylaxis

**Figure 6:**
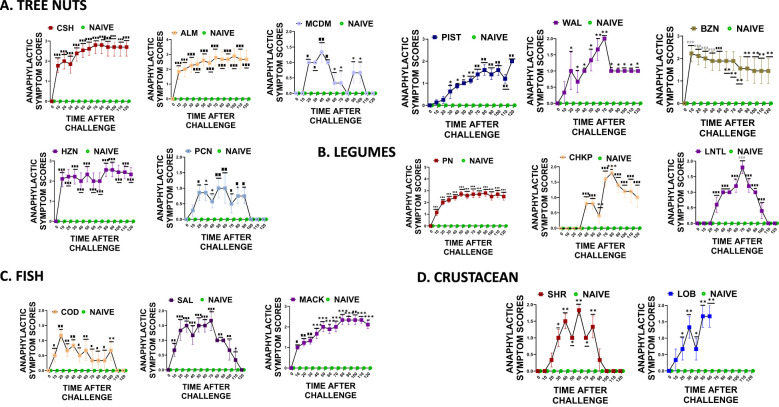
Anaphylactic symptom scores. At weeks 4-5, mice received intragastrical (i.g.) challenges (200 mg/mouse of extract) as follows: **(A)** Tree nuts such as Cashew, Almond, Macadamia, Pistachio, Walnut, Brazil nut, Hazelnut, and Pecan. **(B)**. Legumes such as Peanut, Chickpea, and Lentil. **(C)** Fish such as Cod, Salmon, and Mackerel **(D)**. Crustaceans such as Shrimp and Lobsters. Anaphylactic symptom scores were assessed every 10 mins till 120 mins following each food challenge. *p< 0.05; **p< 0.01; ***p< 0.001 vs sham. n=10-15 mice/group.

During weeks 5-6 of the study, intragastric oral food challenges were conducted using 200mg of freshly prepared peanut, cashew, almond, pistachio, walnut, pecan, shrimp, lobster, cod, and salmon. The mice were scored for anaphylactic symptoms as per the previously described methods. Our findings indicate that mice sensitized with peanut, cashew, walnut, shrimp, and cod protein showed anaphylactic symptoms to cross-reactive proteins such as chickpea, lentil, almond, pistachio, hazelnut, brazil nut, pecan, lobster, salmon, and mackerel. The mice that were sensitized and then given oral peanut food allergens showed significantly higher anaphylactic symptom scores compared to the naive mice that did not undergo sensitization (**Figure 6B** * *p*< 0.05; ** *p*< 0.01; ***p< 0.001 vs sham). Additionally, mice that received cross-reactive food allergens from the legume food group, such as chickpea and lentil, also exhibited significantly higher anaphylactic symptom scores compared to the naive group (**Figure 6B** * *p*< 0.05; ** *p*< 0.01; ***p< 0.001 vs sham). Similar trends were observed in the tree nut food group, where mice that were previously sensitized and then exposed to cashew and walnut showed significantly higher anaphylactic symptom scores, (**Figure 6A** * *p*< 0.05; ** *p*< 0.01; ***p< 0.001 vs sham). Furthermore, significantly higher anaphylactic symptom scores were observed in mice exposed to cross-reactive tree nut allergens like brazil nut, almond, pistachio, pecan, and macadamia (**Figure 6A** * *p*< 0.05; ** *p*< 0.01; ***p< 0.001 vs sham). The fish and crustacean group also displayed similar patterns, with mice exposed to both cod and shrimp allergens showing significantly higher anaphylactic symptom scores, along with mice exposed to salmon, mackerel, and lobster, despite not being sensitized to these allergens (**Figures 6C, D** * *p*< 0.05; ** *p*< 0.01; ***p< 0.001 vs sham).

### 3.6 Cross-reactive food allergens induced the release of plasma histamine

**Figure 7:**
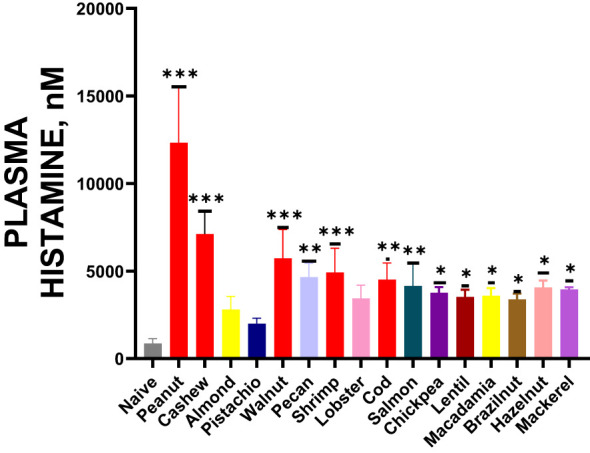
Histamine Release: After completing the temperature measurement, blood was drawn by tail-vein nick, plasma was harvested, and histamine levels were measured by ELISA. Inoculated foods have red solid bars. *p< 0.05; **p< 0.01; ***p< 0.001 vs sham. n=10-15 mice/group.

During the oral food challenge, we collected blood samples by making a small incision in the tail vein. From these samples, we extracted plasma to measure histamine levels using the ELISA method. Our analysis showed increased plasma histamine levels following exposure to cross-reactive allergens, as illustrated in **Figure 7** * *p*< 0.05; ** *p*< 0.01; ***p< 0.001 vs sham.

## 4 Discussion

The phenomenon of cross-reactivity among certain food allergens is widely recognized, but there is a lack of effective treatment options for food allergies. Researchers are actively seeking better treatment options. Currently, limited animal models are available for studying potential therapies for cross-reactive food allergens. Our study represents the first attempt to establish an animal model designed to investigate cross-reactive food allergens. We showed IgE-mediated cross-reactivity across the major food allergens.

In this study, we created an animal model to investigate cross-reactivity with various food groups, including legumes, tree nuts, fish, and shellfish, using the C3HeJ mouse. Over three weeks, we injected the mice intraperitoneally with at least one allergen from each food group. After this sensitization phase, we measured the levels of IgE using ELISA. In this study for the oral challenge, we administered a dose of 500 µg of allergen with 2 mg of alum, based on previously established models of food allergy using C3H/HeJ mice (23). This dose is consistent with those reported in other murine models that exhibit IgE responses and symptoms of anaphylaxis (23). Moreover, this dose provides significant allergen exposure that can lead to cross-reactivity, which is the focus of our study aimed at understanding cross-reactivity among multiple food groups. We believe our findings are in line with other studies since the current dose elicited strong anaphylactic responses (23).

In our research, we found that the mice showed increased levels of IgE in response to the sensitized food and similar or even higher levels of IgE in response to other foods with similar allergens. Specifically, we sensitized C3HeJ mice with peanuts from the legume food group and measured their IgE levels for peanuts and other legumes like lentils and chickpeas. The levels of peanut-specific IgE were consistent with previous studies, particularly those by Yang et al. (20) and Verma et al. (25)). Verma et al. (25) also reported similar levels of IgE in chickpea-sensitized mice. However, there have been no reports of cross-reactive IgE to chickpeas and lentils in a mouse model. In general, we observed a rise in IgE levels in response to sensitizing food groups, including tree nuts, fish, and seafood, similar to the increase in the legume food groups. When we sensitized the mice with cashews, walnuts, shrimp, and cod via intraperitoneal injection, we noted elevated IgE levels in reaction to the sensitizing food allergens as well as to cross-reactive challenge foods such as almond, pistachio, hazelnut, brazil nut, pecan, lobster, salmon, and mackerel. To the best of our knowledge, this is the first study to report an increase in IgE to cross-reactive food allergens across major food allergen groups.

Using the ELISA method, this study quantifies IgE, IgG1, and IgG2a antibodies. However, direct quantification is challenging due to the absence of homologous standards for each allergen. Although the ELISA values presented in this study indicate relative levels, they provide significant insights into the IgE and IgG responses within our model and yield valuable data regarding cross-reactive allergenic responses. Previous research conducted by Neil et al. (26) used an ELISA-based method to measure food-specific IgE antibodies in mouse serum. They demonstrated that it was effective to coat the ELISA plates with food extracts at 10 to 5000 micrograms per milliliter concentrations in a carbonate buffer. Similarly, we applied this method in our study and coated the ELISA plates for measuring allergen-specific IgE.

Sera from mice immunized with alum contains a high amount of IgG antibodies, which makes it difficult to measure allergen-specific immunoglobulin levels. To measure allergen-specific IgE in sera with high IgG, we adopted methods from previous research. Specifically, we referenced the study by Yang et al. (20), which investigated the inhibition of pathological immunoglobulin E (IgE) in food allergies using EBF-2 and the active compound berberine, both of which are associated with immunometabolism regulation. In this study, peanut-specific IgE levels were measured using a detection antibody, IgE Biotin Rat Anti-Mouse IgE, in conjunction with Purified Rat Anti-Mouse IgE as the capture antibody. This approach was used to assess allergen-specific IgE in the sera of C3HeJ mice that had been immunized with alum. According to the manufacturer, BD Biosciences, the rat anti-mouse IgE antibody is specifically reactive with mouse IgE and has been reported to not interact with other immunoglobulin isotypes. Following this established method from prior publications, we assessed allergen-specific IgE in the sera of our mice immunized with alum.

In this study, alum was used as an adjuvant during the sensitization phase of our study. Adjuvants are crucial in immunology studies because they enhance immune responses, resulting in a more robust and detectable reaction to allergens (27). In this study, alum likely intensified the IgE response and facilitated cross-reactivity among food allergens. Including alum in the study impacts the interpretation of results in several ways. The strong immune response triggered by alum enables the simulation of heightened allergenic reactions, which may be more intense than some natural human responses. Therefore, the findings reflect a maximized immune response valuable for studying potential therapeutic interventions but may not fully replicate the allergenic responses in humans without an adjuvant. Alum’s adjuvant effects could exaggerate the allergenic response, leading to higher IgE levels and more pronounced anaphylactic symptoms than would typically occur in a model without an adjuvant. This heightened response ensures that cross-reactive allergies are detectable but may influence the perceived severity of cross-reactivity among the tested allergens. While using alum is essential for achieving significant results in the murine model, the findings should be interpreted with caution regarding their applicability to human allergic responses. The impact of adjuvants like alums means that the model is highly effective for studying cross-reactivity patterns and potential interventions, but it may not completely represent milder human allergies. As such, the alum’s role in this study aids in effectively modeling cross-reactivity among multiple food allergens while necessitating careful consideration in interpreting results for real-world human allergic responses.

In this study, histamine measurements were chosen over mouse mast cell protease-1 (MMCP-1) to assess allergic responses, particularly to evaluate the severity of anaphylactic reactions in the murine model. Histamine is a direct marker of mast cell degranulation and is known to play a pivotal role in immediate allergic responses, which makes it a highly relevant biomarker for assessing anaphylaxis. Histamine release is a critical mediator of type I hypersensitivity reactions, including anaphylaxis. Elevated plasma histamine levels are directly associated with vasodilation, increased vascular permeability, and smooth muscle contraction, all hallmark signs of anaphylaxis. By measuring histamine, we directly assessed the extent and severity of the allergic response in real-time, providing a clear picture of systemic anaphylaxis following allergen challenges​. Histamine has a rapid onset and is detectable shortly after mast cell degranulation, making it an ideal marker for capturing the immediate effects of allergen exposure. Since this study involved repeated allergen challenges, histamine’s quick release allowed us to measure peak responses accurately, especially when aiming to observe acute allergic responses across various food allergens​. Histamine is commonly measured in human studies to evaluate allergic reactions and is clinically relevant as an indicator of allergic severity. By choosing histamine as a marker, the study findings can more readily align with human clinical observations, enhancing the model’s translational relevance for potential therapeutic interventions​.

In a study conducted by Cox et al. (10), they found varying prevalence rates of legume allergy and different patterns of cross-sensitization and clinical cross-reactivity to various legumes in humans. While numerous studies report sensitization to different foods, there is a lack of challenge-proven clinical allergy in humans to these different foods. However, in our study, we were able to demonstrate challenge-proven allergy to chickpeas and lentils in the allergic peanut-allergic mice, as well as challenge-proven allergy to almonds, pistachios, hazelnuts, brazil nuts, and pecans in the tree nut-sensitized mice and to lobster in the shellfish allergic group and salmon and mackerel in the fish allergic group. Our results indicated comparable anaphylactic symptom scores in mice exposed to food allergens that were not previously sensitized.

In murine models of food allergy, the core body temperature decreases following repeated exposure to the allergens, indicating anaphylaxis. Measuring core body temperature is a practical and dependable way to assess allergic reactions. It allows for easy evaluation of strategies to prevent or treat allergic reactions (28). It can also be used to assess the severity of anaphylaxis and between different food allergens across different groups. In this study, we observed a drop in rectal temperature, a clinical measure of anaphylaxis, in response to a food challenge with the relevant cross-reactive food allergens. Notably, our study is the first to report an increase in IgE levels to cross-reactive food allergens across major food allergen groups. These findings provide valuable insights into clinical reactivity to allergens and can be used as an investigative tool to measure clinical reactivity in cases of *in vitro* sensitization.

Smaldini and colleagues (29) investigated *in vivo* evidence of cross-reactivity between cow’s milk and soybean proteins using a mouse model of food allergy. They demonstrated that there is cross-reactivity between these two protein sources in a murine model. Their study showed that mice sensitized to cow’s milk protein, without prior exposure to soybean proteins, exhibited signs of hypersensitivity immediately after the oral administration of soy protein. This finding suggests that immunochemical cross-reactivity could have clinical relevance. Additionally, we identified cross-reactivity among various allergen groups, such as legumes and tree nuts, using a murine model. While Smaldini’s research focused on only two proteins, our study examines cross-reactivity across a wider range of allergens, including defatted extracts of peanuts, tree nuts, shellfish, and fish. This approach provides a more comprehensive understanding of food allergen cross-reactivity.

The research conducted by Yamamoto et al. (30) identifies tropomyosin as an IgE cross-reactive protein linking house dust mite (HDM) and coho salmon, which may play a role in the development of salmon allergy. In their murine model, they demonstrated that tropomyosin serves as an IgE cross-reactive protein between HDM and coho salmon, highlighting the potential for salmon allergy to occur following an HDM allergy. Our findings support the concept of IgE-mediated cross-reactivity; however, our study differs by focusing on cross-allergic reactions among various food groups instead of between food and non-food allergens. Our model offers a unique perspective by broadening the assessment of allergens and cross-reactivity beyond the typical priority food allergens (PFAS) to include different food groups such as legumes and tree nuts (Yamamoto et al. 30).

Vinje et al. (31) (2012) investigated cross-allergic reactions to legumes in mice sensitized to lupin and fenugreek. The researchers explored the cross-reactivity among different legumes and found that mice sensitized to lupin and fenugreek exhibited cross-allergy to peanut, soy, fenugreek, and lupin. The differences observed in serological responses between primary allergy and cross-allergy may result from different immune mechanisms or variations in epitope affinity to IgE. Our findings align with Vinje and colleagues’ results regarding IgE cross-reactivity within legumes and tree nuts, such as peanuts and cashews. However, our study goes further by examining cross-reactivity across a broader range of allergen groups beyond legumes, demonstrating the potential applicability of the model for various food allergies.

Smeekens et al. (32) developed a mouse model to study the cross-reactivity between shrimp, crab, and lobster in cases of seafood allergies. Their research demonstrated the presence of shrimp-specific IgE that is also cross-reactive with both crab and lobster, leading to anaphylactic reactions upon exposure to shrimp. Similarly, our model investigates cross-reactivity within seafood by examining allergic reactions to both shellfish and fish allergens. This provides evidence of cross-reactive allergic responses among crustaceans and fish. Our approach aligns with the findings of Smeekens et al., but it expands the analysis by including tree nuts and legumes, thereby addressing a gap in the current literature on cross-reactivity.

### 4.1 Limitations

One potential limitation of this study is its exclusive focus on cross-reactive food allergens in a single mouse model, specifically the C3HeJ mice. To address this limitation in future research, it would be valuable to incorporate various mouse models, such as the BalbC, C57BL/6 mice, and other models utilized in *in vivo* studies of food allergy and anaphylaxis. This broader approach would enhance the comprehensiveness and applicability of our findings.

In this study we utilized defatted food extracts instead of purified proteins. While these extracts are complex and contain non-allergenic components, they were chosen to mimic a broader exposure to allergenic whole foods. This approach aligns with previously published research. For example, Pons et al. (24) explored soy immunotherapy for peanut-allergic mice, focusing on modulating the peanut-allergic response. In their study, crude peanut extract (CPE) and crude soybean extract (CSE) were both prepared from defatted raw flours. Similarly, a study by Li et al. (23) described a murine model of peanut anaphylaxis, demonstrating that the T- and B-cell responses to a major peanut allergen closely mimic the human response. In this case, defatted crude peanut extracts were used as allergens to sensitize the mice. Our study adopted the same method as seen in previous studies, sensitizing the mice with defatted food extracts. This approach allows for the assessment of cross-reactivity in a manner that reflects real-life dietary habits, offering insights into allergic responses to whole foods rather than isolated proteins. Future studies might consider using purified proteins to conduct more targeted investigations into specific allergenic components.

## 5 Conclusions

We have successfully developed a comprehensive murine model to study IgE-mediated anaphylactic reactions caused by multiple food groups with cross-reactivity. This model is a crucial step forward in research to develop new and effective treatments for individuals suffering from cross-reactive multiple food allergies.

## Data Availability

The original contributions presented in the study are included in the article/**Supplementary Material**. Further inquiries can be directed to the corresponding author.
