# Supplementary material for: Murine model of cross-IgE sensitization and cross-anaphylactic reactions among multiple group food allergens
Source: Front Immunol. 2025 Jan 7;15:1497368. doi: 10.3389/fimmu.2024.1497368 (PMC11753245; doi:10.3389/fimmu.2024.1497368)
Supplement: Supplementary file 1 [file Supplementaryfile1.pptx]

## Slide 1
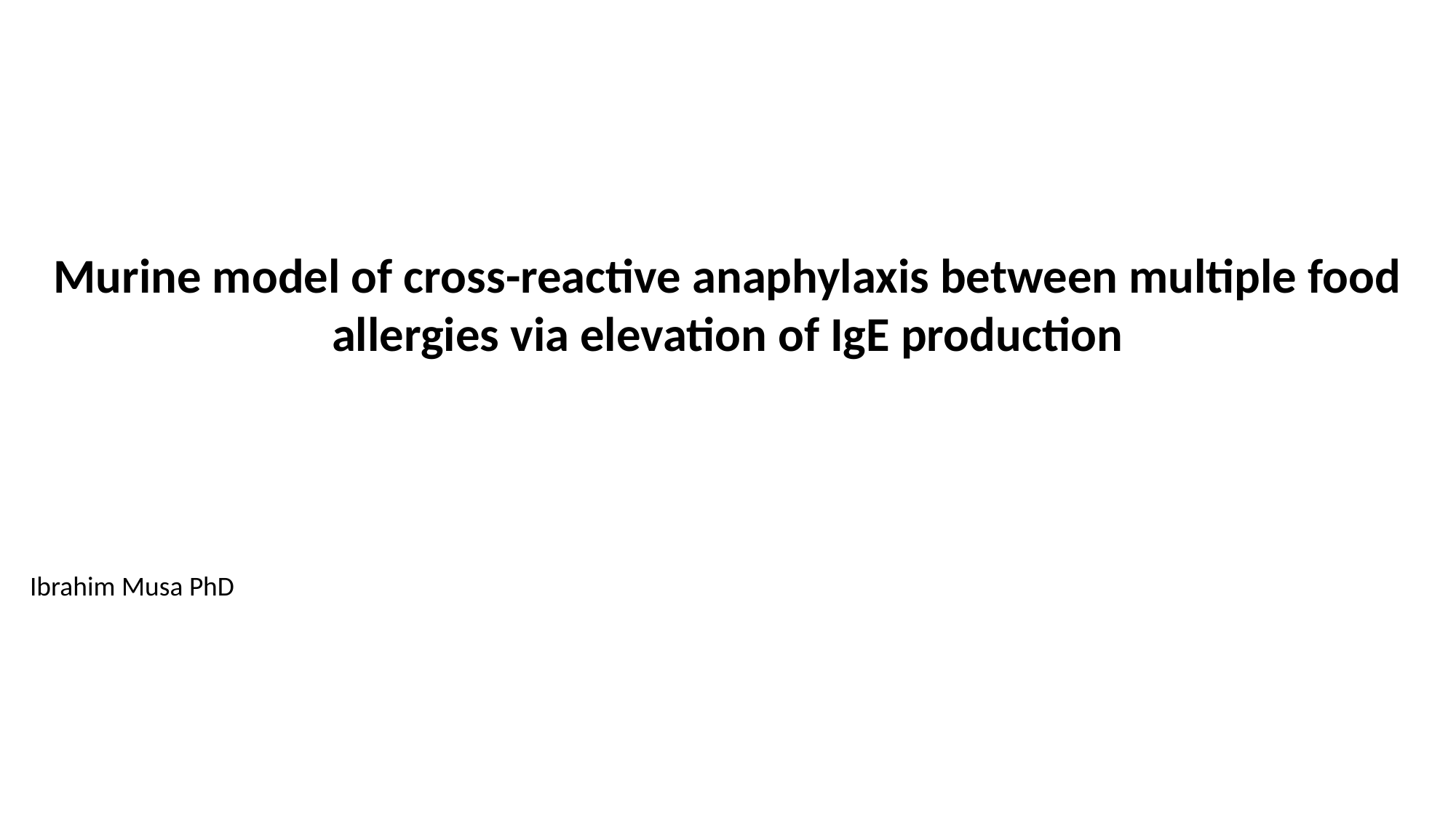

Murine model of cross-reactive anaphylaxis between multiple food allergies via elevation of IgE production
Ibrahim Musa PhD

## Slide 2
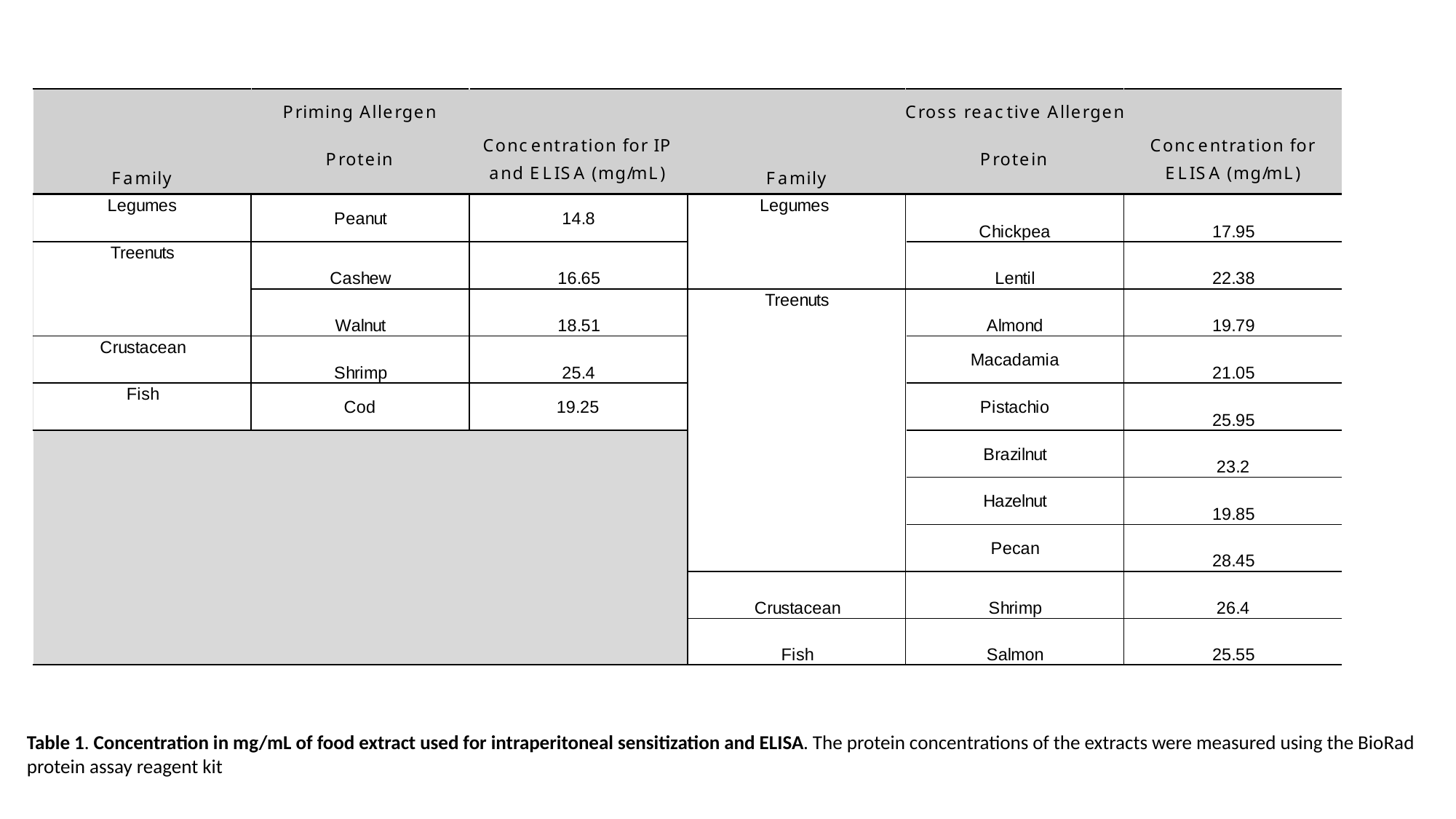

Table 1. Concentration in mg/mL of food extract used for intraperitoneal sensitization and ELISA. The protein concentrations of the extracts were measured using the BioRad protein assay reagent kit

## Slide 3
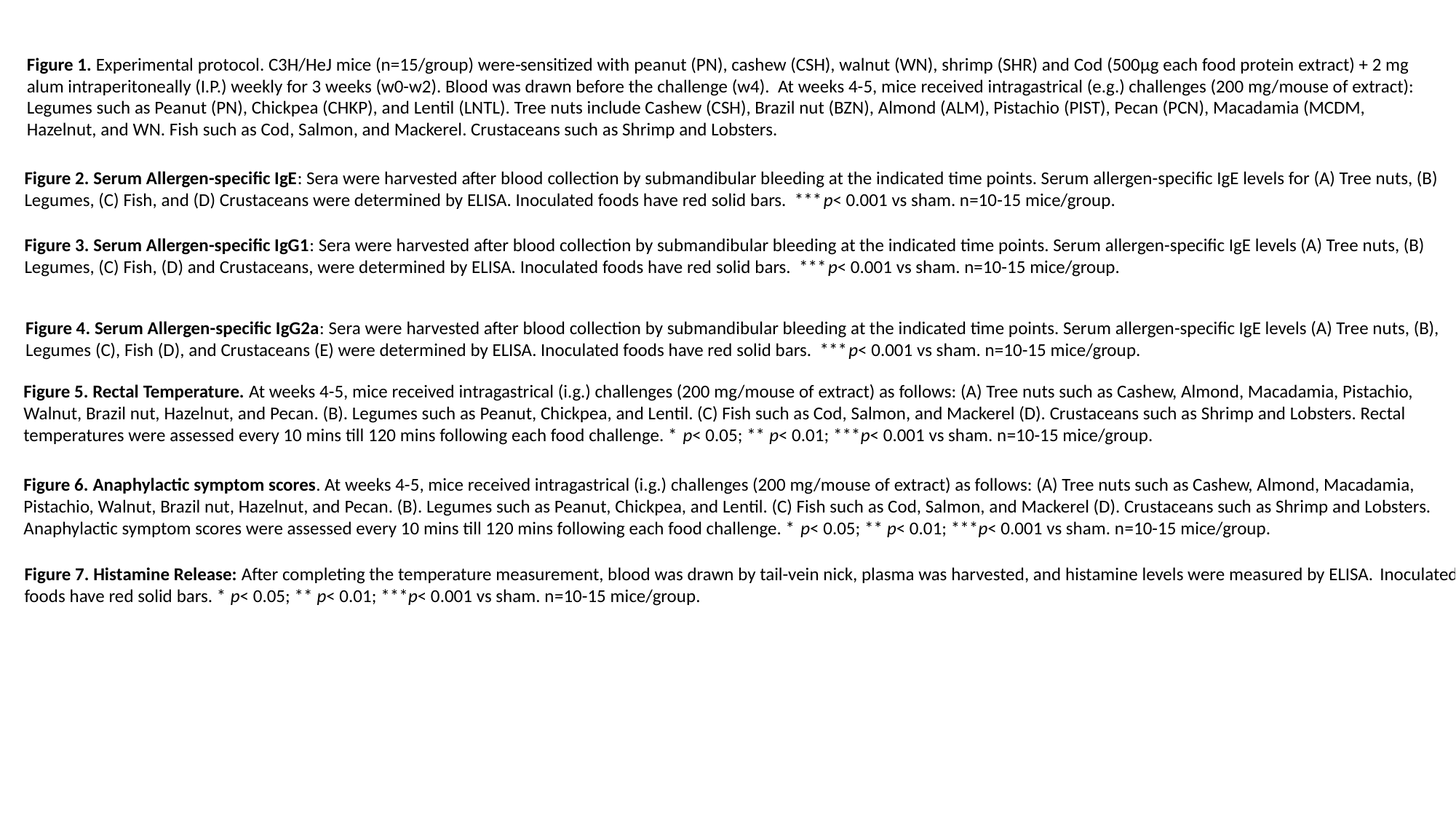

Figure 1. Experimental protocol. C3H/HeJ mice (n=15/group) were sensitized with peanut (PN), cashew (CSH), walnut (WN), shrimp (SHR) and Cod (500µg each food protein extract) + 2 mg alum intraperitoneally (I.P.) weekly for 3 weeks (w0-w2). Blood was drawn before the challenge (w4). At weeks 4-5, mice received intragastrical (e.g.) challenges (200 mg/mouse of extract): Legumes such as Peanut (PN), Chickpea (CHKP), and Lentil (LNTL). Tree nuts include Cashew (CSH), Brazil nut (BZN), Almond (ALM), Pistachio (PIST), Pecan (PCN), Macadamia (MCDM, Hazelnut, and WN. Fish such as Cod, Salmon, and Mackerel. Crustaceans such as Shrimp and Lobsters.
Figure 2. Serum Allergen-specific IgE: Sera were harvested after blood collection by submandibular bleeding at the indicated time points. Serum allergen-specific IgE levels for (A) Tree nuts, (B) Legumes, (C) Fish, and (D) Crustaceans were determined by ELISA. Inoculated foods have red solid bars. ***p< 0.001 vs sham. n=10-15 mice/group.
Figure 3. Serum Allergen-specific IgG1: Sera were harvested after blood collection by submandibular bleeding at the indicated time points. Serum allergen-specific IgE levels (A) Tree nuts, (B) Legumes, (C) Fish, (D) and Crustaceans, were determined by ELISA. Inoculated foods have red solid bars. ***p< 0.001 vs sham. n=10-15 mice/group.
Figure 4. Serum Allergen-specific IgG2a: Sera were harvested after blood collection by submandibular bleeding at the indicated time points. Serum allergen-specific IgE levels (A) Tree nuts, (B), Legumes (C), Fish (D), and Crustaceans (E) were determined by ELISA. Inoculated foods have red solid bars. ***p< 0.001 vs sham. n=10-15 mice/group.
Figure 5. Rectal Temperature. At weeks 4-5, mice received intragastrical (i.g.) challenges (200 mg/mouse of extract) as follows: (A) Tree nuts such as Cashew, Almond, Macadamia, Pistachio, Walnut, Brazil nut, Hazelnut, and Pecan. (B). Legumes such as Peanut, Chickpea, and Lentil. (C) Fish such as Cod, Salmon, and Mackerel (D). Crustaceans such as Shrimp and Lobsters. Rectal temperatures were assessed every 10 mins till 120 mins following each food challenge. * p< 0.05; ** p< 0.01; ***p< 0.001 vs sham. n=10-15 mice/group.
Figure 6. Anaphylactic symptom scores. At weeks 4-5, mice received intragastrical (i.g.) challenges (200 mg/mouse of extract) as follows: (A) Tree nuts such as Cashew, Almond, Macadamia, Pistachio, Walnut, Brazil nut, Hazelnut, and Pecan. (B). Legumes such as Peanut, Chickpea, and Lentil. (C) Fish such as Cod, Salmon, and Mackerel (D). Crustaceans such as Shrimp and Lobsters. Anaphylactic symptom scores were assessed every 10 mins till 120 mins following each food challenge. * p< 0.05; ** p< 0.01; ***p< 0.001 vs sham. n=10-15 mice/group.
Figure 7. Histamine Release: After completing the temperature measurement, blood was drawn by tail-vein nick, plasma was harvested, and histamine levels were measured by ELISA. Inoculated foods have red solid bars. * p< 0.05; ** p< 0.01; ***p< 0.001 vs sham. n=10-15 mice/group.
